# Sleep macro- and microstructure in breast cancer survivors

**DOI:** 10.1038/s41598-022-06664-z

**Published:** 2022-02-15

**Authors:** J. Perrier, M. Duivon, P. Clochon, S. Rehel, F. Doidy, J. M. Grellard, C. Segura-Djezzar, J. Geffrelot, G. Emile, D. Allouache, C. Levy, S. Polvent, F. Viader, F. Eustache, F. Joly, B. Giffard

**Affiliations:** 1 Normandie Univ, UNICAEN, PSL Université, EPHE, INSERM, U1077, CHU de Caen, GIP Cyceron, Neuropsychologie Et Imagerie de La Mémoire Humaine, 14000 Caen, France; 2 Departments of Clinical Research Unit and Medical Oncology, Caen, France; 3 Institut Normand du Sein, Centre François Baclesse, Caen, France; 4 CHU Côte de Nacre, Caen, France; 5 INSERM, Normandie Univ, UNICAEN, U1086 ANTICIPE, Caen, France; 6 Cancer and Cognition Platform, Ligue Nationale Contre Le Cancer, 14076 Caen, France

**Keywords:** Cancer, Neurophysiology

## Abstract

Complaints of sleep disturbance are prevalent among breast cancer (BC) patients and are predictors of quality of life. Still, electrophysiological measures of sleep are missing in patients, which prevents from understanding the pathophysiological consequences of cancer and its past treatments. Using polysomnography, sleep can be investigated in terms of macro- (e.g. awakenings, sleep stages) and micro- (i.e. cortical activity) structure. We aimed to characterize sleep complaints, and macro- and microstructure in 33 BC survivors untreated by chemotherapy and that had finished radiotherapy since at least 6 months (i.e. out of the acute effects of radiotherapy) compared to 21 healthy controls (HC). Compared to HC, BC patients had a larger number of awakenings (*p* = 0.008); and lower Delta power (*p* < 0.001), related to sleep deepening and homeostasis; greater both Alpha (*p* = 0.002) and Beta power (*p* < 0.001), related to arousal during deep sleep; and lower Theta power (*p* = 0.004), related to emotion regulation during dream sleep. Here we show that patients have increased cortical activity related to arousal and lower activity related to sleep homeostasis compared to controls. These results give additional insights in sleep pathophysiology of BC survivors and suggest sleep homeostasis disruption in non-advanced stages of BC.

## Introduction

Breast cancer (BC) is the most frequently diagnosed in women in the majority of countries worldwide^1^ with greatest prevalence of sleep complaints, notably insomnia ones, compared to other cancer types^2,3^. Insomnia is defined as a dissatisfaction with sleep quality caused by difficulties to falling asleep, maintaining sleep, early morning awakening, or non-restorative sleep leading to daytime impairments, at least three times a week and for at least 3 months^4^. Sleep complaints are now well recognized in non-central nervous system (CNS) cancer patients, not only following chemotherapy^5,6^ but also before the occurrence of treatments^7–11^. Despite significant sleep complaint from patients^12,13^, it has not been fully attested by objective evaluations such as polysomnography (PSG) that is considered as the gold standard of sleep evaluation^14,15^. Previous experimental studies dedicated to evaluate sleep using PSG in BC patients are scarce and have shown either a lack of sleep alterations following chemotherapy^16–18^ or deleterious effects of chemotherapy and/or radiotherapy^19,20^. Regarding radiotherapy, one study reported larger duration of NREM Stage 2 following radiotherapy in breast cancer patients compared to healthy controls^19^. However, in this study, the delay between the PSG recording and the end of radiotherapy was unclear.

PSG allows to go further in the understanding of sleep modifications, not only in its macrostructure (i.e. sleep architecture as reflected by sleep stages, sleep efficiency, total sleep time, number of awakening), but also in its microstructure as reflected by spectral analyses of the electroencephalography (EEG). Especially, power spectrum analyses consist of computing Fast Fourier Transforms on EEG signal in order to reveal the frequency and the amplitude of its waves. Results are combined by averaging the data in different frequency bands that have been previously related to sleep electrophysiological modifications. For examples, sleep spectral analyses provide additional information related to deepening of sleep (i.e. Delta band), to emotion and dreaming (i.e. Theta band)^21^, to sleep fragility and environmental awareness (i.e. Alpha band)^22,23^, or to arousal particularly during Non-Rapid Eye Movement—NREM sleep (i.e. Beta band)^24^. With healthy ageing, previous reports have shown that Delta power decreases and Alpha and Sigma power increase, indicating that sleep becomes lighter with age^25,26^. In insomnia disorder, a recent meta-analysis reported increased Alpha, Sigma and Beta power and lower Delta power compare to healthy adults, notably during NREM sleep^27^.

In the context of cancer, it may be that, despite subtle modification in sleep architecture, alterations in the sleep electroencephalogram can be more visible using spectral analysis of sleep as previously shown in patients suffering from insomnia disorder. Indeed, in these patients suffering from insomnia disorder, without other comorbidities such as sleep-apnea or cancer, subjective complaints of sleep disturbance assessed by self-rating questionnaires are often discordant with sleep measured objectively using PSG^28^. In contrast, greater cortical arousal (i.e. increased Beta power spectrum) has been successfully revealed during sleep in insomnia using spectral analyses^24^. Indeed, as in aging, sleep spectral analysis can thus be a more sensitive measure of sleep disruption than sleep architecture^29^, making such analysis potentially useful in highlighting slight sleep disruptions and allowing deepen understanding of electrophysiological modifications associated with sleep in BC patients. To our knowledge, no study until now has investigated sleep spectral analysis in non-CNS cancers patients. Still, previous reports suggested that tumors disrupt the homeostatic processes of sleep^30^, a process that is related to the Delta power spectrum during sleep. Sleep is well-known to be under the regulation of two processes (i.e. S and C), that interact continuously^31^. Process S refers to sleep pressure accumulated during the day, while process C is related to circadian rhythms over 24 h.

The current cross-sectional study aimed at quantifying sleep macro- and microstructure modifications with PSG in BC survivors untreated by adjuvant chemotherapy and evaluated at least 6 months after radiotherapy (i.e. out of the acute effects of radiotherapy) compared to healthy controls. Overall, we expected greater sleep complaints and sleep structure modifications in BC patients compared to healthy controls.

## Results

### Participant’s characteristics

Demographics and psychological characteristics of HC and patients and clinical characteristics of patients are presented in Table 1. No significant difference was observed between groups for demographics and psychological characteristics.

**Table 1:** Demographical and psychological characteristics of HC and BC patients, and clinical characteristics of patients (means and standard deviations).

	HC (n = 21)	BC (n = 33)	Group effects (HC vs BC)
	Means (SDs)		*p*-values
Age (years)	62.6 (4.4)	60.2 (5.4)	0.62
Education (years)	11.8 (1.7)	12.4 (3.5)	0.49
Depression (BDI)	4.1 (3.2)	3.0 (2.4)	0.39
Trait anxiety (STAI)	42.3 (9.3)	40.6 (9.6)	0.53
Stage of the cancer		*N (%)*	
0 I IIA	NA NA NA	17 (52%) 12 (36%) 4 (12%)	NA
Breast-conserving surgery Mastectomy	NA NA	29 (88%) 5 (15%)*	NA
Time since radiotherapy (months)	NA	8.0 (2.4) 5 not treated	NA

HC: Healthy Controls; BC: Breast cancer patients; BDI: Beck Depression Inventory; STAI: State-Trait Anxiety Inventory; NA: Not Applicable; * one patient had a breast-conserving surgery and a mastectomy; Wilcoxon-Mann–Whitney test was realized.

### Sleep disturbance complaints

Results of subjective sleep disturbance are reported Table 2. BC patients had significantly more sleep complaints (*M* = 8.1, *SD* = 4.5) and insomnia symptoms (*M* = 11.8, *SD* = 6.5) than HC (PSQI: *M* = 5.5, *SD* = 2.8, *p* = 0.012, ISI: *M* = 7.2, *SD* = 4.5, *p* = 0.004). According to the ISI, 63% of BC patients and 48% of HC had complaints of sleep disturbance related to insomnia (i.e. ISI score ≥ 8). This rate of participants having sleep disturbance did not differ significantly between the two groups (*p*=0.43).

**Table 2:** Subjective sleep complaints of HC and BC patients.

	HC (n = 21)	BC (n = 32*)	Group effects (HC vs BC)	
	Means (SDs)		*t(df=52)*	*p*-values
Sleep quality (PSQI)	5.5 (2.8)	8.1 (4.5)	2.61	**0.012**
Insomnia severity (ISI)	7.2 (4.5) ISI < 8: n=11 ISI ≥ 8: n=10	11.8 (6.5) ISI < 8: n=12 ISI ≥ 8: n=20	3.03 X-squared = 4.90	**0.004** 0.43

*One 1 patient whose questionnaires answered at home are missing.

HC: Healthy Controls; BC: Breast cancer patients; PSQI: Pittsburgh Sleep Quality Index; ISI: Insomnia Severity Index. Student’s t test was realized. Pearson chi-squared test was used to determine whether sleep disturbance rate varied between groups. *p*-values<0.05 are in bold.

### Sleep macrostructure (sleep architecture) results

Sleep architecture measures are reported in Table 3. A significant group effect was observed on the number of awakenings longer than 1 min with BC patients having significantly more awakenings (*M* = 9.6, *SD* = 4.3) than HC (*M* = 6.8, *SD* = 2.8, *p* = 0.008).

**Table 3:** Sleep architecture of HC and BC patients (means and standard deviations).

Sleep parameters	HC (n = 21)	BC (n = 33)	Group effects (HC vs BC)		
	Means (SDs)		*F (1,51)*	*p*-values	*η*^*2*^
Time in bed (min)	461.8 (52)	478.4 (50)	1.35	0.25	0.026
Sleep onset latency (min)	24.9 (20)	27.0 (18)	0.15	0.70	0.003
Sleep latency to Stage 3	42.3 (21)	54.8 (40)	1.69	0.20	0.031
Latency to REM (min)	118.8 (54)	121.8 (51)	0.04	0.84	<0.001
Total sleep time (min)	355.5 (68)	353.8 (57)	0.01	0.92	<0.001
Sleep efficiency (%)	76.9 (11)	74.2 (11)	0.73	0.40	0.014
Number of awakenings < 1 min	21.3 (11)	25.1 (13)	1.63	0.21	0.023
Number of awakenings > 1 min	6.8 (2.8)	9.6 (4.3)	7.58	**0.008**	0.13
Number of stage transition	151.4 (47)	164.9 (53)	1.28	0.26	0.017
TST stage 1% Time (min)	8.6 (3.7) 29.7 (12)	8.8 (5.1) 30.8 (18)	0.08 0.072	0.78 0.79	<0.001 <0.001
TST Stage 2% Time (min)	50.3 (7.2) 177.0 (34)	51.9 (6.0) 184.3 (40)	0.78 0.49	0.38 0.49	0.015 0.009
TST Stage 3% Time (min)	22.9 (6.1) 82.5 (31)	22.5 (7.2) 79.1 (27)	0.04 0.20	0.84 0.65	<0.001 0.004
TST stage REM % Time (min)	18.3 (4.8) 66.3 (24)	16.8 (4.0) 59.6 (18)	1.50 1.38	0.23 0.25	0.028 0.026
Apnea hypopnea index (AHI)	19.6 (13)	19.6 (11)	w=360	0.82	NA

HC: Healthy Controls; BC: Breast cancer patients; TST: Total Sleep Time; ANOVA analyses were realized using AHI as a covariate; *p*-values<0.05 are in bold; small (η^2^ ≥ 0.01), medium (η^2^ ≥ 0.06), and large (η^2^ ≥ 0.14) effects.

No significant difference between BC patients and HC was observed neither on other sleep parameters (i.e. TIB, SOL, TST, SE), nor on the percentage of sleep stages.

### Sleep microstructure results

Results are described in Table 4. No significant interaction was observed between factors group and cortical area, thus only group and cortical area effects as well as averages means and standard deviations across all EEG electrodes are reported in Table 4.

**Table 4:** Average power spectra of HC and BC patients (means and standard deviations of all EEG electrodes) according to each sleep stage and frequency band.

Sleep stages	Frequency bands	HC (n = 21)	BC (n = 33)	Group effects (HC vs BC)			Cortical area effects (HC vs BC)		
		Means (SDs)*		*F(1,316)*	*p*	*η*^*2*^	*F(5,316)*	*p*	*η*^*2*^
1	Delta	39.9 (5.1)	37.9 (6.3)	9.31	**0.002**	0.013	76.96	**<0.001**	0.54
	Theta	24.0 (4.9)	23.2 (4.8)	2.11	0.15	0.004	37.25	**<0.001**	0.37
	Alpha	18.7 (4.7)	20.6 (5.7)	10.48	**0.001**	0.026	15.90	**<0.001**	0.20
	Sigma	2.63 (0.9)	2.84 (1.1)	3.62	0.058	0.010	6.29	**<0.001**	0.09
	Beta	13.9 (4.2)	14.8 (4.4)	3.36	0.068	0.009	7.68	**<0.001**	0.11
2	Delta	52.7 (5.8)	50.8 (5.9)	8.27	**0.004**	0.009	111.95	**<0.001**	0.63
	Theta	23.0 (3.3)	22.6 (3.5)	1.02	0.31	0.001	124.82	**<0.001**	0.66
	Alpha	14.4 (4.0)	15.8 (4.9)	7.79	**0.006**	0.020	13.44	**<0.001**	0.17
	Sigma	2.58 (1.3)	2.67 (1.2)	0.37	0.55	0.000	15.82	**<0.001**	0.20
	Beta	8.45 (3.0)	9.30 (2.8)	6.69	**0.010**	0.015	23.89	**<0.001**	0.27
3	Delta	67.0 (5.8)	64.1 (5.9)	18.77	**<0.001**	0.024	89.43	**<0.001**	0.56
	Theta	19.8 (2.6)	20.4 (3.1)	2.96	0.086	0.002	197.36	**<0.001**	0.75
	Alpha	8.94 (3.5)	10.4 (4.2)	9.86	**0.002**	0.028	4.02	**0.002**	0.058
	Sigma	1.31 (0.8)	1.45 (0.7)	2.80	0.095	0.006	27.08	**<0.001**	0.30
	Beta	3.63 (1.4)	4.36 (1.6)	16.73	**<0.001**	0.033	33.43	**<0.001**	0.33
REM	Delta	37.3 (5.6)	36.4 (6.1)	1.53	0.22	0.005	20.39	**<0.001**	0.24
	Theta	25.8 (4.2)	24.2 (4.4)	8.45	**0.004**	0.023	9.88	**<0.001**	0.13
	Alpha	19.2 (3.8)	20.2 (4.6)	3.07	0.081	0.007	19.99	**<0.001**	0.24
	Sigma	2.84 (0.9)	2.95 (1.0)	0.87	0.35	0.002	6.71	**<0.001**	0.10
	Beta	15.5 (5.2)	16.5 (5.6)	2.12	0.15	0.006	2.91	**0.014**	0.04

*Means and standard deviations averaged for all electrodes (FP1/FP2/F3/F4/F7/F8/Fz/C3/C4/Cz/T3/T4/P3/P4/O1/O2) and all stages (Stages 1, 2, 3 and REM sleep). HC: Healthy Controls; BC: Breast cancer patients; ANOVA analyses were realized using AHI as a covariate; *p*-values<0.05 are in bold;; small (η^2^ ≥ 0.01), medium (η^2^ ≥ 0.06), and large (η^2^ ≥ 0.14) effects. Groups of electrodes were averaged together to increased statistical power into prefrontal (FP1/FP2), frontal (F3/F4/F7/F8/Fz), central (C3/C4/Cz), temporal (T3/T4), parietal (P3/P4/Pz), and occipital (O1/O2) areas. Statistical analyses were performed using AHI as a covariate. No significant interaction was found between cortical area and group effects; thus, these results are not reported.

During Stages 1 and 2, power spectra for the Delta (*ps* < 0.01) and Alpha (*ps* < 0.01) frequency bands differed between groups. BC patients had significantly lower Delta (Stage 1: *M* = 37.9, *SD* = 6.3, Stage 2: *M* = 50.8, *SD* = 5.9) than HC (Stage 1: *M* = 39.9, *SD* = 5.1, *p* = 0.002, Stage 2: *M* = 52.7, *SD* = 5.8, *p* = 0.004). BC patients also had higher Alpha power spectrum (Stage 1: *M* = 20.6, *SD* = 5.7, Stage 2: *M* = 15.8, *SD* = 4.9) than HC (Stage 1: *M* = 18.7, *SD* = 4.7, *p* = 0.001, Stage 2: *M* = 14.4, *SD* = 4.0, *p* = 0.006). In addition, during Stage 2, Beta power differed between groups with BC patients having higher Beta power (*M* = 9.30, *SD* = 2.8) than HC (*M* = 8.45, *SD* = 3.0, *p* = 0.01).

During Stage 3, a significant effect of group was observed for Delta, Alpha, and Beta frequency bands (*ps* < 0.01). BC patients had lower Delta (*M* = 64.1, *SD* = 5.9) and higher Beta (*M* = 4.36, *SD* = 1.6) and Alpha (*M* = 10.4, *SD* = 4.2) power spectrum than HC (Delta: *M* = 67.0, *SD* = 5.8, *p* < 0.001, Beta: *M* = 3.63, *SD* = 1.4, *p* < 0.001, Alpha: *M* = 8.94, *SD* = 3.5, *p* = 0.002).

During REM sleep, Theta power spectrum differed between groups, with BC patients having lower Theta power (*M* = 24.2, *SD* = 4.4) than HC (*M* = 25.8, *SD* = 4.2, *p* = 0.004).

## Discussion

The current cross-sectional study aimed at quantifying sleep macro- and microstructure modifications with PSG in BC survivors untreated by adjuvant chemotherapy and evaluated at least 6 months after radiotherapy compared to HC. We focused not only on sleep architecture but also on more fine-grained investigations related to sleep (i.e. spectral analyses) based on EEG signal and thus reflecting cortical effects. These results are close to those reported in healthy ageing and insomnia disorder, which questioned about the contribution of insomnia symptoms and age to these modifications. Still, because our groups did not differ in terms of insomnia symptoms rate and in terms of age, we can suppose that it reflects an effect of cancer and its past treatments. To our knowledge, this is the first study to report spectral analyses results during sleep in BC patients, allowing a deepen and finer understanding of sleep pathophysiology in BC patients.

Regarding sleep architecture, results revealed greater number of awakenings in BC patients than in HC. Regarding spectral analyses of cortical EEG during sleep, results revealed modifications of power spectrum in Delta, Alpha and Beta bands during NREM sleep (i.e. Stages 1, 2 and 3) in BC patients compared to HC. During REM sleep, BC patients differed from HC for the Theta power spectrum. Of note, no significant topographical difference was highlighted between patients and controls suggesting that the main sources of sleep related EEG activity did not differ between groups but was rather a more widespread effect over the scalp.

Despite a lack of significant difference in sleep efficiency (i.e. time in bed/total sleep time), BC patients had larger number of awakenings (longer than one minute) than HC. These results suggest a more fragmented sleep with short arousals in BC patients that did not affect sleep efficiency. Overall, BC patients seem to have subtle but still present modifications of sleep architecture compared to HC. These results are in accordance with those of previous studies suggesting effects of cancer and/or diagnostic on sleep^32,33^. One may argue that the greater number of awakenings in patients could be driven by the effects of radiotherapy as most of our sample of patients was treated with radiotherapy following surgery. However, we ensure a period of at least 6 months between the end of radiotherapy and the PSG recordings in order to avoid acute effects of radiotherapy on sleep. In addition, previous studies that have measured sleep, either with PSG or actigraphy, following radiotherapy did not report significantly larger number of awakenings related to radiotherapy either compared to before radiotherapy or compared to HC^34,35^. Although further studies are needed to confirm this result, we may assume that sleep modifications reported in the current study are not specifically related to the long-term effects of radiotherapy.

Regarding sleep microstructure, BC patients had lower Delta power spectrum than HC during NREM sleep. Previous reports in rats model of non-metastatic BC have shown Delta power reduction^36^ that was accompanied by aberrant activity of hypocretin/orexin neurons that are involved in the sleep/wake cycle regulation. Therefore, our results confirm previous findings and help to understand pathophysiology of BC during sleep. The Delta band during NREM sleep depends on the precise timing of thalamo-cortical activity^37^ and is known to reflect the homeostatic process of sleep (i.e. process S, where amplitude of Delta activity during NREM increases relative to the duration of prior waking)^31,38^. These results give rationale for altered activity in brain circuits regulating sleep homeostasis (i.e. hypocretin/orexin neurons) in BC patients. These results thus support a previous hypothesis about tumor disrupting sleep homeostasis in rodents’ model of non-metastatic breast cancer^30^. This result is also in accordance with a previous report suggesting sleep homeostasis disruption in advanced stages BC patients (stages III and IV)^20^. It may thus be possible that sleep homeostasis is disrupted in BC, even in non-advanced stages as represent by the current sample (i.e. mostly stages 0 and I).

This result is in line with recent hypotheses proposing that tumors disrupt homeostatic processes. In addition, the current results suggest less deepen sleep in BC patients with potential deleterious effects on daytime functioning. BC patients had increase in the Alpha power spectrum during NREM sleep, referring to the Alpha-Delta sleep. As NREM sleep deepened, Alpha disappears and the re-appearance of Alpha during slow-wave sleep (Stage 3) has been associated with non-restorative sleep^39^. Alpha during NREM sleep has also been associated with cortical hyperarousal in insomnia patients^40^. Seemly, the greater Beta power in BC patients compared to HC during NREM sleep suggests greater cortical arousal in BC patients. This greater cortical arousal could be related to non-restorative sleep as the Beta EEG band has been previously linked to cortical arousal during sleep in insomnia patients (e.g.^27,40–43^). Greater cortical hyperarousal in insomnia has been previously linked to greater risk for developing depressive symptoms^44^, highlighting the need for further light over sleep disturbance in BC patients in order to prevent depressive symptoms. During REM sleep, BC patients had reduction of the Theta power spectrum compared to HC. The Theta band is the central activity of REM sleep and has been associated with dreaming and emotion^21,45^, suggesting alteration in emotion regulation related to sleep in BC patients. Still, this hypothesis remains to be tested.

### Limitations

This study is an exploratory one with a small number of participants, further studies using larger sample sizes are warranted. Nevertheless, it paves the road for further investigation in order to better understand the pathophysiological modifications associated with BC and its treatments.

## Conclusions

Altogether, our results highlighted modifications of sleep structure in BC patients compared to HC. BC patients had a greater number of awakenings during sleep as well as greater arousal and a less deepen sleep than HC during NREM sleep as reflected by cortical activity. During REM sleep, BC patients seem to have deregulation of EEG bands related to emotional regulation and dreaming. Although we had a small sample size, our results give additional insights in sleep pathophysiology of BC survivors and suggest sleep homeostasis disruption in non-advanced stages of BC.

## Materials and methods

### Participants

All participants provided written informed consent for the study. The study was conducted according to the guidelines of the Declaration of Helsinki, and approved by the ethics committee (CPP Ile de France III), n°ID-RCB: 2017-A02778-45.

Participants were recruited between January 2018 and March 2020 at the François Baclesse regional cancer center (Caen, France). Among the patients approached, 50 agreed to participate. Eleven patients finally declined to participate. An additional six patients were removed from the analyses due to PSG recording issues (i.e. holter shut down prematurely) during the ambulatory home-based PSG. Among the 25 healthy controls (HC) included, four finally refused to participate. Thus, groups consisted of 33 patients and 21 HC. Informed consent was obtained from all subjects involved in the study.

Patient inclusion criteria were : (i) less than 70 years old, (ii) no metastatic BC, (iii) already undergone surgical or radiotherapy treatment, (iv) radiotherapy finished since at least six months ago and no chemotherapy treatment, (v) menopausal status since at least one year ago at the time of inclusion, (vi) no personality disorder nor progressive psychiatric disorder (vii) no neurological sequelae, (viii) no drug use or alcohol abuse, (ix) be a native French speaker, (x) have at least a primary school level of education.

Inclusion criteria for HC were the same as for patients, including no history of cancer and no global cognitive impairment according to the Montreal Cognitive Assessment. HC were matched in age and education with BC patients.

### Measures

#### Questionnaires

Depression was assessed with the Beck Depression Inventory (BDI)^46^ and anxiety was measured with the State Trait Anxiety Inventory (STAI)^47^. For both questionnaires higher values indicate higher depression and trait-anxiety symptoms. The STAI questionnaire was not fulfilled at the same time that sleep measurement, thus the State score was not representative of their anxiety state at the time of sleep and only Trait anxiety score was reported here.

Perceived sleep quality was assessed using the Pittsburgh Sleep Quality Index (PSQI), a 19-item questionnaire measuring sleep habits (e.g. sleep latency, frequency of awakenings) over the previous month. Scores range from 0 to 21, with higher scores reflecting poorer sleep quality. Insomnia’s symptoms were measured with the Insomnia Severity Index (ISI) composed of seven items evaluating difficulty falling asleep, maintaining sleep and the frequency of early morning awakenings, as well as the degree of dissatisfaction with current sleep and its impact on daily activities. Scores range from 0 to 28, with higher scores indicating higher insomnia related symptoms. The interpretation of the total score is as follows: lack of insomnia (0–7); sub-threshold insomnia (8–14); moderate insomnia (15–21); and severe insomnia (22–28)^48^. The cut-off of 8 has been validated in cancer patients^49,50^, representing the best specificity and sensitivity ratio in this population.

#### Polysomnography

A full night of sleep was recorded using ambulatory PSG at home. Twenty electroencephalography (EEG) electrodes were placed over prefrontal (FP1/FP2), frontal (F3/F4/F7/F8/Fz), central (C3/C4/Cz), temporal (T3/T4), parietal (P3/P4/Pz), and occipital (O1/O2) sites, according to the international 10–20 system. Ag/Au electrodes with a vertex ground and a bi-mastoids reference were used. Two electrodes were placed at each place: above and under the eyes, on the chin, and under each clavicle. This montage included thoracic and abdominal belts, nasal and oral thermistors, nasal airflow and a finger pulse oximeter. All sensors were connected to the Compumedics Siesta sleep system and placed by an EEG technician (SP). The hardware EEG filter band pass was 0.15–121 Hz and the sample rate was set to 256 Hz. Sleep stages (1, 2, 3, and REM) were visually scored by an electrophysiology technician (SR) in 30 s epochs according to the AASM Scoring Manual procedures^51^ using Compumedics software. Artifacts were detected visually by JP, and epochs with artifacts (eye-movement, electrocardiogram, electromyogram or movement-related artifacts) were rejected from analyses. Sleep onset latency (SOL, min) was determined as the time from lights out to the first epoch (i.e. 30 s) of Stage 2 sleep, according to AASM Scoring Manual^51^. Then, sleep architecture parameters included: total time in bed (TIB, min), total sleep time (TST, min), sleep efficiency (SE, %), latency to Stage 3 (min), number of awakenings, number of stage transition, time (min) and percentage (%, according to TST) of Stages 1, 2, 3 and rapid eye movement (REM). AHI (Apnea–Hypopnea Index) is defined as the sum of apneas and hypopneas (with 3% desaturation or arousal) divided by the total sleep time in hours. This index has been visually scored by a sleep technician (SR) according to AASM criteria^51^.

#### Spectral analysis

Computerized spectral analysis was performed with fast Fourier transformation (FFT) on the all-night filtered EEG. Spectral analysis was performed on 4-s epochs and on the 17 channels recorded to investigate the difference in areas EEG power spectra. Before computing the FFT, the data were tapered with the Hamming window. The FFT was computed on artifact-free epochs. The FFT was realized for each sleep stage on the total number of epochs corresponding to the maximal number of artifact-free epochs observed in all subjects. The relative power spectrum was obtained in the following frequency bands: Delta (1.5–4 Hz); Theta (4–7.5 Hz); Alpha (7.5–12.5 Hz); Sigma (12.5–14 Hz); Beta (14–30 Hz). All night power averages were obtained for Stages 1, 2, 3 and REM sleep separately. For spectral analyses, groups of electrodes were averaged together to increased statistical power into prefrontal (FP1/FP2), frontal (F3/F4/F7/F8/Fz), central (C3/C4/Cz), temporal (T3/T4), parietal (P3/P4/Pz), and occipital (O1/O2) areas.

### Statistical methods

Analyses were performed with R software, with statistical significance set at *p* < 0.05. The nonparametric Wilcoxon-Mann–Whitney test was used instead of Student’s t test, if the normality hypothesis was rejected according to Shapiro–Wilk test, to evaluate whether demographic, psychological and subjective sleep differed between groups. Pearson chi-squared test was used to determine whether sleep disturbance rate differed between BC patients and HC.

We performed ANOVA to evaluate whether architecture parameters differed between groups, with sleep architecture parameters as dependent variable, group as the independent variable and AHI as co-variable. To evaluate whether the sleep microstructure differed between groups and across cortical areas, we realized an ANOVA for each frequency band (i.e. Delta, Theta, Alpha, Sigma and Beta) in each sleep stage (i.e. Stages 1, 2 and 3 and REM sleep) with EEG power as dependent variable, group and cortical areas (i.e. prefrontal, frontal, central, temporal, parietal, and occipital areas) as independent variables and AHI as co-variable. Effect size were calculated using eta squared values^52,53^. Effects sizes were considered as follows: η^2^, small effect: η2 ≥ 0.01, medium effect: η2 ≥ 0.06, and large effect: η2 ≥ 0.14^54^.

## Data Availability

Data will be provided upon reasonable request to the corresponding author (JP).

## References

[1] Ferlay J (2019). Estimating the global cancer incidence and mortality in 2018: GLOBOCAN sources and methods. Int. J. Cancer.

[2] Otte JL, Wu J, Yu M, Shaw C, Carpenter JS (2016). Evaluating the sleep hygiene awareness and practice scale in midlife women with and without breast cancer. J. Nurs. Meas..

[3] Savard J, Ivers H, Villa J, Caplette-Gingras A, Morin CM (2011). Natural course of insomnia comorbid with cancer: An 18-month longitudinal study. J. Clin. Oncol..

[4] Morin CM (2015). Insomnia disorder. Nat. Rev. Disease Primers.

[5] Lowery-Allison AE (2018). Sleep problems in breast cancer survivors 1–10 years posttreatment. Palliat Support Care.

[6] Palesh O (2008). Vagal regulation, cortisol, and sleep disruption in women with metastatic breast cancer. J. Clin. Sleep Med..

[7] Chen D, Yin Z, Fang B (2018). Measurements and status of sleep quality in patients with cancers. Support. Care Cancer.

[8] Fox RS (2019). Sleep disturbance and cancer-related fatigue symptom cluster in breast cancer patients undergoing chemotherapy. Support. Care Cancer.

[9] Fox RS (2020). Sleep disturbance and cancer-related fatigue symptom cluster in breast cancer patients undergoing chemotherapy. Support. Care Cancer.

[10] Savard J, Morin C (2001). Insomnia in the context of cancer: A review of a neglected problem. J. Clin. Oncol..

[11] Van Onselen C (2013). Trajectories of sleep disturbance and daytime sleepiness in women before and after surgery for breast cancer. J. Pain Symptom Manag..

[12] Accortt EE, Bower JE, Stanton AL, Ganz PA (2015). Depression and vasomotor symptoms in young breast cancer survivors: the mediating role of sleep disturbance. Arch. Womens Ment. Health.

[13] Fortner BV, Stepanski EJ, Wang SC, Kasprowicz S, Durrence HH (2002). Sleep and quality of life in breast cancer patients. J. Pain Symptom Manag..

[14] Kushida CA (2001). Comparison of actigraphic, polysomnographic, and subjective assessment of sleep parameters in sleep-disordered patients. Sleep Med.

[15] Van De Water ATM, Holmes A, Hurley DA (2011). Objective measurements of sleep for non-laboratory settings as alternatives to polysomnography—a systematic review. J. Sleep Res..

[16] Silberfarb PM, Hauri PJ, Oxman TE, Schnurr P (1993). Assessment of sleep in patients with lung cancer and breast cancer. J. Clin. Oncol..

[17] Roscoe JA (2011). Few changes observed in polysomnographic-assessed sleep before and after completion of chemotherapy. J. Psychosom. Res..

[18] Aldridge-Gerry A (2013). Psychosocial correlates of sleep quality and architecture in women with metastatic breast cancer. Sleep Med..

[19] Tag Eldin E-S, Younis SG, Aziz LMAE, Eldin AT, Erfan ST (2019). Evaluation of sleep pattern disorders in breast cancer patients receiving adjuvant treatment (chemotherapy and/or radiotherapy) using polysomnography. J. Buon..

[20] Parker KP (2008). Sleep/Wake patterns of individuals with advanced cancer measured by ambulatory polysomnography. J. Clin. Oncol..

[21] Scarpelli, S., Bartolacci, C., D’Atri, A., Gorgoni, M. & De Gennaro, L. The functional role of dreaming in emotional processes. Front. Psychol. **10** (2019). 10.3389/fpsyg.2019.00459 PMC6428732 30930809

[22] Simor P, Horváth K, Ujma PP, Gombos F, Bódizs R (2013). Fluctuations between sleep and wakefulness: wake-like features indicated by increased EEG alpha power during different sleep stages in nightmare disorder. Biol Psychol.

[23] McKinney SM, Dang-Vu TT, Buxton OM, Solet JM, Ellenbogen JM (2011). Covert waking brain activity reveals instantaneous sleep depth. PLoS ONE.

[24] Feige B (2013). The microstructure of sleep in primary insomnia: an overview and extension. Int. J. Psychophysiol..

[25] Yoon J-E (2021). Sleep structure and electroencephalographic spectral power of middle-aged or older adults: Normative values by age and sex in the Korean population. J Sleep Res.

[26] Hertenstein E (2018). Reference data for polysomnography-measured and subjective sleep in healthy adults. J. Clin. Sleep Med..

[27] Zhao W (2021). EEG spectral analysis in insomnia disorder: A systematic review and meta-analysis. Sleep Med Rev.

[28] Rezaie L, Fobian AD, McCall WV, Khazaie H (2018). Paradoxical insomnia and subjective-objective sleep discrepancy: A review. Sleep Med Rev.

[29] Edinger JD, Ulmer CS, Means MK (2013). Sensitivity and specificity of polysomnographic criteria for defining insomnia. J. Clin. Sleep Med..

[30] Walker, W. H. & Borniger, J. C. Molecular mechanisms of cancer-induced sleep disruption. *Int. J. Mol. Sci.* **20** (2019). 10.3390/ijms20112780 PMC6600154 31174326

[31] Borbély AA, Daan S, Wirz-Justice A, Deboer T (2016). The two-process model of sleep regulation: a reappraisal. J. Sleep Res..

[32] Ancoli-Israel S (2014). Sleep, fatigue, depression, and circadian activity rhythms in women with breast cancer before and after treatment: a 1-year longitudinal study. Support. Care Cancer.

[33] Beverly CM (2018). Change in longitudinal trends in sleep quality and duration following breast cancer diagnosis: Results from the Women’s Health Initiative. NPJ Breast Cancer.

[34] Dhruva A (2012). A longitudinal study of measures of objective and subjective sleep disturbance in patients with breast cancer before, during, and after radiation therapy. J. Pain Symptom Manag..

[35] Tag Eldin E-S, Younis SG, Aziz LMAE, Eldin AT, Erfan ST (2019). Evaluation of sleep pattern disorders in breast cancer patients receiving adjuvant treatment (chemotherapy and/or radiotherapy) using polysomnography. J. BUON.

[36] Borniger JC (2018). A role for hypocretin/orexin in metabolic and sleep abnormalities in a mouse model of non-metastatic breast cancer. Cell Metab..

[37] Contreras D, Timofeev I, Steriade M (1996). Mechanisms of long-lasting hyperpolarizations underlying slow sleep oscillations in cat corticothalamic networks. J Physiol.

[38] Borbély AA (1982). A two process model of sleep regulation. Hum. Neurobiol..

[39] Krystal AD, Edinger JD, Wohlgemuth WK, Marsh GR (2002). NREM sleep EEG frequency spectral correlates of sleep complaints in primary insomnia subtypes. Sleep.

[40] Riedner BA (2016). Regional patterns of elevated alpha and high-frequency electroencephalographic activity during nonrapid eye movement sleep in chronic insomnia: A pilot study. Sleep.

[41] Perlis M, Pigeon W, Gehrman P, Findley J, Drummond S (2009). Neurobiological mechanisms in chronic insomnia. Sleep Med. Clin..

[42] Perrier J (2015). Specific EEG sleep pattern in the prefrontal cortex in primary insomnia. PLoS ONE.

[43] Riemann D (2015). The neurobiology, investigation, and treatment of chronic insomnia. Lancet Neurol..

[44] Baglioni C (2011). Insomnia as a predictor of depression: a meta-analytic evaluation of longitudinal epidemiological studies. J. Affect. Disord..

[45] Boyce R, Glasgow SD, Williams S, Adamantidis A (2016). Causal evidence for the role of REM sleep theta rhythm in contextual memory consolidation. Science (New York, NY).

[46] Beck AT, Ward CH, Mendelson M, Mock J, Erbaugh J (1961). An inventory for measuring depression. Arch. Gen. Psychiatry.

[47] Spielberger CD, Gorsuch RL, Lushene RE (1970). The state-trait anxiety inventory (test manual).

[48] Morin CM, Belleville G, Bélanger L, Ivers H (2011). The insomnia severity index: Psychometric indicators to detect insomnia cases and evaluate treatment response. Sleep.

[49] Savard M-H, Savard J, Simard S, Ivers H (2005). Empirical validation of the Insomnia Severity Index in cancer patients. Psychooncology.

[50] Michaud AL, Zhou ES, Chang G, Recklitis CJ (2021). Validation of the Insomnia Severity Index (ISI) for identifying insomnia in young adult cancer survivors: Comparison with a structured clinical diagnostic interview of the DSM-5 (SCID-5). Sleep Med..

[51] Berry RB (2017). AASM scoring manual updates for 2017 (Version 2.4). J. Clin. Sleep Med..

[52] Lakens, D. Calculating and reporting effect sizes to facilitate cumulative science: A practical primer for t-tests and ANOVAs. Front. Psychol. **4** (2013). 10.3389/fpsyg.2013.00863 PMC3840331 24324449

[53] Levine TR, Hullett CR (2002). Eta squared, partial eta squared, and misreporting of effect size in communication research. Hum. Commun. Res..

[54] Cohen, J. *Statistical Power Analysis for the Behavioral Sciences. Hillsdle*. (Erlbaum. Conner, BE (1988). The Box in the Barn. Columbus: Highlights for …, 1988).

